# Analysis of the immunologic status of a newly diagnosed HIV positive population in China

**DOI:** 10.1186/1471-2334-13-429

**Published:** 2013-09-11

**Authors:** Yinzhong Shen, Hongzhou Lu, Zhenyan Wang, Tangkai Qi, Jiangrong Wang

**Affiliations:** 1 Department of Infectious Diseases, Shanghai Public Health Clinical Center, Fudan University, Shanghai 201508, China

**Keywords:** Human immunodeficiency virus, CD4^+^T lymphocyte count, China

## Abstract

**Background:**

The immunologic status of a newly diagnosed HIV positive population in the era of antiretroviral therapy in China has not been extensively evaluated. We conducted a cross-sectional survey to evaluate the CD4 counts of newly diagnosed HIV-infected persons and determine the factors influencing these counts in China.

**Methods:**

Two thousand eight hundred and sixty-six newly diagnosed HIV-infected patients from 10 provinces in China were selected during 2009 to 2010. Serum samples were collected to measure CD4 counts by flow cytometry. Demographics and medical histories were recorded. Multivariate logistic regression models were used to analyze factors associated with low CD4 count (<100 cells/mm^3^) at HIV diagnosis.

**Results:**

Among the 2866 patients, 2159 (75.33%) were male. Mean age was 40 years (range: 18–86 years). The median CD4 count at HIV diagnosis was 83 cells/mm^3^, 72.02% of the patients had a CD4 count that was ≤200 cells/mm^3^, 53.98% had CD4 counts <100 cells/mm^3^. The difference in CD4 counts between males and females was not statistically significant (*P*=0.469). The median CD4 count differed significantly according to age (*P*=0.002), province (*P*<0.001), ethnicity (*P*<0.001) and HIV transmission route (*P*<0.001). In multivariate logistic analysis, factors associated with greater odds of low CD4 count at HIV diagnosis included male sex, younger age, HIV transmission route classified as unknown transmission risk, having been diagnosed in provinces Guangxi, Henan, Heilongjiang, Jiangxi, Shanghai and Yunnan.

**Conclusions:**

At the time of HIV diagnosis, a large proportion of HIV-infected patients in China had an initial CD4 count that was consistent with relatively advanced disease. Low CD4 count was associated with male gender, younger age, route of HIV transmission and geographical areas. HIV testing policy that promotes routine testing for HIV infection is needed to facilitate earlier HIV diagnosis.

## Background

HIV/AIDS has become one of the major public health problems in China. Although the overall prevalence of HIV remains relatively low in China, the epidemic is characterized by geographical disparities and a higher prevalence reported among certain sub-groups [1]. In November of 2003, the Chinese Ministry of Health announced a national policy of free antiretroviral treatment (ART) to all HIV positive Chinese citizens who were in poverty and required ART. The free ART has improved HIV-infected patients’ quality of life, prolonged patients’ lives, and reduced the AIDS mortality rate [2,3]. Delayed diagnosis of HIV is associated with a worse prognosis despite ART. China has boosted its screening efforts for HIV, with an increase in the number of persons diagnosed with HIV [4]. Despite the enormous attention being paid to early diagnosis and treatment of HIV/AIDS worldwide, studies still show that most patients present late for care [5-7].

HIV infection leads to immune system collapse, following a gradual destruction of CD4^+^T lymphocytes that causes a severe immune suppression and consequently a high risk of opportunistic infections. All HIV-infected patients should have a baseline CD4 count measured at entry into care [8]. The CD4 count is the major laboratory indicator of immune function in HIV-infected patients [8]. It is a useful tool to guide the initiation of ART and prophylaxis for opportunistic infections, and it is the strongest predictor of subsequent disease progression and survival [9,10]. In China, HIV diagnosis is mostly done in severe or acute clinical circumstances, or among high-risk groups, blood donors, or within voluntary counseling and testing (VCT) facilities. In order to learn about the immunologic status of a newly diagnosed HIV positive population in China, we conducted a cross-sectional survey to evaluate the baseline CD4 counts of newly diagnosed HIV-infected persons and investigate the factors related to low CD4 level at HIV diagnosis.

## Methods

### Study population

This study was part of the research content of the 12th Five-Year infectious disease research program which was supported by the Ministry of Science and Technology, China. We conducted a cross-sectional survey on HIV/AIDS in China’s HIV epidemic provinces including Shaanxi, Xinjiang, Hunan, Guangdong, Yunnan, Heilongjiang, Shanghai, Henan, Jiangxi, and Guangxi during 2009 to 2010. The survey subjects were newly diagnosed HIV/AIDS patients who had not received ART. The patients were addressed to the HIV diagnosis and management hospitals, because they had symptoms and or risky behaviors. Patients aged 18 years or more at the time of enrolment with documented HIV-1 infection were eligible for this study. Only antiretroviral naïve patients were included in the study. Being on ART was an exclusion criterion. Antiretroviral experienced but currently untreated patients were not eligible for this study. All patients were confirmed to be positive for HIV antibody through laboratory detection, and the diagnosis was in line with national HIV/AIDS diagnostic criteria. Demographic characteristics and medical histories of the patients were recorded.

### Blood samples

For newly diagnosed HIVAIDS patients, two-milliliter venous blood samples were obtained from each individual at the initial visit in order to make a CD4 count. Written informed consent was obtained for all subjects; consent forms and procedures, as well as survey protocol, were approved by the Shanghai Public Health Clinical Center Ethics Committee. CD4 count was measured by flow cytometry at the clinical laboratories in each province. All the study laboratories successfully completed a standardization and certification program. The study doctors provided comprehensive care for the study patients according to the guidelines for diagnosis and treatment of HIV/AIDS in China before and after the blood test was done.

### Data collection

Data were collected according to standardised criteria. On enrolment, standardised data collection forms were completed at the sites providing information from patient interview and patient case notes. Data collected on newly identified cases included demographic information, risk-behavior information (injection drug use, sexual risk behavior, receipt of blood transfusion), and laboratory test results. Variables of interest included province (geographical area of HIV diagnosis), age, sex, ethnicity, and HIV transmission route. Age was denoted as ≤24, 25–34, 35–44, 45–54 or ≥55 years. Race/ethnicity was designated as Han, Zhuang, Uygur or other. HIV transmission route was categorized as sexual contact (including homosexual or heterosexual, the data on homosexual sex were not collected, sexual contact was not designated as homosexual or heterosexual sex in this study), blood transfusion, injection drug use, or unknown transmission risk.

### Statistical analysis

SPSS software for Windows (Version 11.5; SPSS Inc., Chicago, IL) was used for statistical analysis. Data were described using median (interquartile range, IQR) and frequencies. We performed Mann–Whitney *U* test or Kruskal-Wallis H test to compare CD4 counts between different subgroups. Multivariate logistic regression models were used to analyze factors associated with low CD4 count at HIV diagnosis. The fitness of the final model was assessed with the likelihood ratio test. Low CD4 count was defined as CD4 count <100 cells/mm^3^ at HIV diagnosis. Factors included in the models were province, sex, age, ethnicity and HIV transmission route. All variables included in the models were determined a priori based on epidemiological importance and biological plausibility. The statistical test was two-tailed and performed at a level of statistical significance of 0.05.

## Results

### Patient characteristics

We investigated a total of 2866 newly diagnosed HIV-infected patients from 10 provinces; 1048 patients were from Guangxi, 529 from Yunnan, 51 from Henan, 222 from Xinjiang, 64 from Jiangxi, 197 from Guangdong, 95 from Shaanxi, 176 from Hunan, 80 from Heilongjiang, and 404 from Shanghai. Table 1 describes the basic characteristics of the study population according to province. Overall, the patients had a male majority (75.33%). The mean age was 40 years (41 years for male, 38 years for female) (range: 18–86 years). Most patients acquired HIV through sexual contact (74.42%).

**Table 1:** Basic characteristics of study HIV/AIDS patients in China (n)

**Characteristics**	**Total**	**Guangdong**	**Guangxi**	**Henan**	**Heilongjiang**	**Hunan**	**Jiangxi**	**Shaanxi**	**Shanghai**	**Xinjiang**	**Yunnan**
Number of patients	2866	197	1048	51	80	176	64	95	404	222	529
Age, years											
18–24	131	20	33	3	5	15	4	9	23	3	16
25–34	941	91	303	11	29	50	16	33	115	98	195
35–44	911	53	301	26	22	60	23	32	114	100	180
45–54	463	18	185	7	14	29	13	14	90	16	77
≥55	420	15	226	4	10	22	8	7	62	5	61
Mean age	40	35	43	40	39	39	41	37	41	36	40
Sex											
Male	2159	144	799	34	64	111	50	70	362	177	348
Female	707	53	249	17	16	65	14	25	42	45	181
HIV transmission category											
Sexual contact	2133	171	936	6	53	119	63	68	330	46	341
IDU	454	8	92	0	3	41	0	7	6	166	131
Blood transfusion	81	3	0	29	17	3	0	12	7	3	7
Unknown	198	15	20	16	7	13	1	8	61	7	50
CD4 count, cells/mm^3^											
<100	1547	69	738	37	40	63	49	17	224	65	245
≥100	1319	128	310	14	40	113	15	78	180	157	284
Median CD4 count	83	159	37	51	101	172	42	285	79	183	112
Ethnicity											
Han	1998	194	444	50	79	176	63	95	401	21	475
Zhuang	593	0	590	0	0	0	0	0	0	0	3
Uygur	183	0	0	0	0	0	0	0	1	181	1
Other	92	3	14	1	1	0	1	0	2	20	50

*IDU* Intravenous drug use.

### Immunologic status of newly diagnosed HIV/AIDS patients

**Figure 1:**
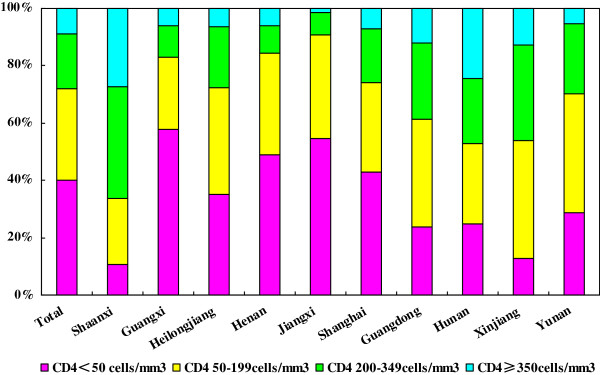
The distribution of CD4 counts in newly diagnosed HIV-infected patients in China by province.

Table 1 and Figure 1 show the immunologic status of newly diagnosed HIV/AIDS patients. The median CD4 count at HIV diagnosis was 83 cells/mm^3^ (IQR, 24–220) (range: 1–926 cells/mm^3^), and the mean was 138 cells/mm^3^. Among the 2866 patients, patients with a CD4 count of <50, 50 to 199, 200 to 349, 350 to 499, and ≥500 cells/mm^3^ accounted for 40.06%, 31.93%, 19.26%, 5.96% and 2.79%, respectively. The patients with CD4 count ≤200 cells/mm^3^ accounted for 72.02%, whereas 53.98% of the patients had a CD4 count that was <100 cells/mm^3^, and only 8.75% of the patients had a CD4 count that was ≥350 cells/mm^3^.

### Immunologic status of HIV/AIDS patients in different provinces

The median CD4 counts of HIV/AIDS patients at diagnosis in 10 provinces are listed in Table 1. The CD4 counts at HIV diagnosis differed significantly according to province (*P*<0.001). The highest and lowest median CD4 counts of patients were found in Shaanxi and Guangxi province, respectively. The proportion of patients with a CD4 count of <100 cells/mm^3^ at HIV diagnosis were: Guangdong 35.03%, Guangxi 70.42%, Henan 72.55%, Heilongjiang 50.00%, Hunan 35.80%, Jiangxi 76.56%, Shaanxi 17.90%, Shanghai 55.45%, Xinjiang 29.28% and Yunnan 46.31%. The distribution of CD4 counts at HIV diagnosis in HIV/AIDS patients in different provinces is shown in Figure 1.

### Immunologic status of HIV/AIDS patients according to sex and age

Table 2 describes the median CD4 counts among HIV/AIDS patients. The median CD4 counts in male and female patients were 81 cells/mm^3^ and 97 cells/mm^3^, respectively. The difference in CD4 counts between males and females was not statistically significant (*P*=0.469). The CD4 count at HIV diagnosis differed significantly according to age (*P*=0.002), the median CD4 count was highest in patients who were 18 to 24 years of age.

**Table 2:** The median CD4 counts among study HIV/AIDS patients

**Cohort**	**Median CD4 count(IQR)(cells/mm**^**3**^**)**
Sex	
Male	81(24, 213)
Female	97(23, 233)
P value for difference*	0.469
Age	
≤24	123(22, 290)
25–34	90(24, 234)
35–44	87(24, 209)
45–54	54(20, 189)
≥55	93(30, 210)
P value for difference#	0.002
Ethnicity	
Han	93(25, 229)
Zhuang	40(16, 127)
Uygur	164(85, 263)
Other	116(30, 227)
P value for difference#	<0.001
HIV transmission category	
Sexual contact	67(21, 202)
Intravenous drug use	163(64, 271)
Blood transfusion	98(32, 209)
Unknown transmission risk	70(25, 205)
P value for difference#	<0.001

NOTE: * Mann–Whitney *U* test was uesd; # Kruskal-Wallis H test was used.

### Immunologic status of HIV/AIDS patients according to ethnicity

**Figure 2:**
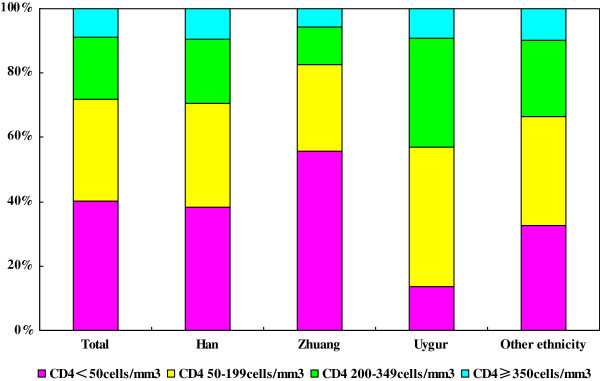
The distribution of CD4 counts in newly diagnosed HIV-infected patients in China by ethnicity.

The CD4 counts at HIV diagnosis differed significantly according to ethnicity (*P*<0.001); the highest and lowest median CD4 count were found in Uygur and Zhuang patients, respectively (Table 2). The distribution of CD4 counts in HIV/AIDS patients with different ethnicities is shown in Figure 2.

### Immunologic status of HIV/AIDS patients with different routes of HIV transmission

**Figure 3:**
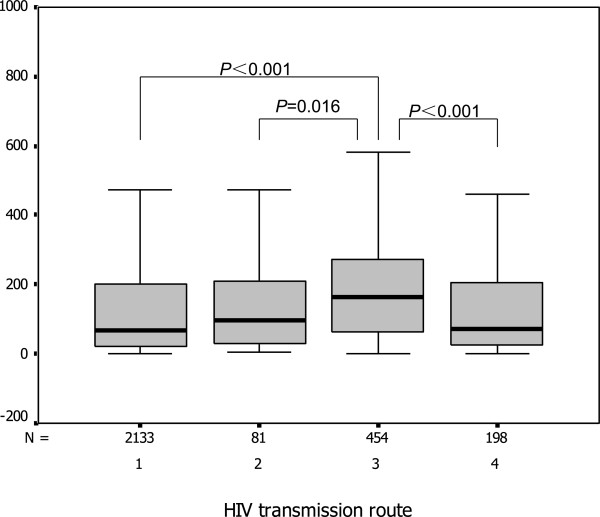
**The CD4 counts in newly diagnosed HIV/AIDS patients with different routes of HIV transmission in China (1: sexual contact, 2: blood transfusion, 3: intravenous drug use, 4: unknown transmission risk.** Each box shows the median, quartiles, and the extreme values within a category) (N: sample size of the group).

The median CD4 counts in HIV patients with different routes of HIV transmission are shown in Table 2. The CD4 count at HIV diagnosis differed significantly according to HIV transmission route (*P*<0.001), the median CD4 count in patients infected with HIV through intravenous drug use was highest (Figure 3).

### Identification of factors associated with low CD4 count at HIV diagnosis

In a multivariate analysis using a multivariate logistic regression model, we analyzed factors (including province, sex, age, ethnicity, HIV transmission route) associated with low CD4 count at HIV diagnosis. Table 3 summarizes the results of the final regression model. In multivariate analysis, factors significantly associated with a lower odds of low CD4 count at HIV diagnosis included HIV transmission route classified as intravenous drug use, older age, and having been diagnosed in Shaanxi province. Factors associated with greater odds of low CD4 count at HIV diagnosis included male sex, HIV transmission route classified as unknown transmission risk, having been diagnosed in provinces Guangxi, Henan, Heilongjiang, Jiangxi, Shanghai and Yunnan. Ethnicity failed to show an association with low CD4 count at HIV diagnosis.

**Table 3:** Identification of factors associated with low CD4 count at HIV diagnosis among newly diagnosed HIV/AIDS patients in China, results of the multivariate logistic regression

Factor*	***P* value**	**Odds ratio**	**95% confidence interval**
Age,per 10-year increment	0.012	0.911	(0.847, 0.980)
Male sex	0.017	1.258	(1.042, 1.518)
Ethnicity	-	-	-
Han	0.280	1.000	-
Zhuang	0.726	0.944	(0.683, 1.303)
Uygur	0.060	2.004	(0.971, 4.134)
Other	0.808	1.065	(0.643, 1.763)
HIV transmission route	-	-	-
Sexual contact	<0.001	1.000	-
Blood transfusion	0.765	1.088	(0.624, 1.898)
Intravenous drug use	<0.001	0.524	(0.405, 0.677)
Unknown transmission risk	0.01	1.518	(1.104, 2.088)
Province			
Guangdong	<0.001	1.000	-
Guangxi	<0.001	5.016	(3.608, 6.972)
Henan	<0.001	4.414	(2.064, 9.440)
Heilongjiang	0.025	1.857	(1.080, 3.193)
Hunan	0.340	1.235	(0.801, 1.903)
Jiangxi	<0.001	6.362	(3.314, 12.210)
Shaanxi	0.004	0.409	(0.223, 0.750)
Shanghai	<0.001	2.251	(1.574, 3.219)
Xinjiang	0.388	1.219	(0.778, 1.909)
Yunnan	<0.001	1.916	(1.353, 2.713)

**NOTE:** *P values of the likelihood ratio test for age, sex, ethnicity, HIV transmission route and province were 0.012, 0.017, 0.850, <0.001 and <0.001, respectively.

## Discussion

The results of the current study reveal that the majority of newly diagnosed HIV positive patients in China had an initial CD4 count consistent with an AIDS diagnosis. The initial CD4 counts of the majority of the patients were already in the range requiring commencement of ART. The results reflect a significant delay in the diagnosis of HIV-infected patients. This delay may compromise early management of HIV-infected individuals and enhances propagation of the epidemic in China.

Prior studies also suggest that a significant proportion of newly diagnosed HIV-infected patients in China are diagnosed late in the course of their disease. A previous investigation [11] conducted in Yunnan and Guangxi showed that the overall median CD4 cell count of the newly identified HIV and AIDS cases in years 2005 to 2009 was 285 cells/mm^3^, and 63% of the patients had CD4 counts <350 cells/mm^3^. Another study [12] showed that the median baseline CD4 cell count of all patients in China national treatment database from June 2002-August 2008 was 118 cells/mm^3^. These findings reinforce results from our study, which show that there are difficulties surrounding the early identification of HIV cases in China.

The results of our study are comparable to some studies in other countries, which also indicate a delay in the diagnosis of HIV infected patients. A study [13] carried out between January 2006 and December 2008 showed that 60.4% of the newly HIV diagnosed Moroccan patients had CD4 counts <200 cells/mm^3^ when HIV diagnosis was confirmed. A similar investigation [14] conducted in Nigeria between January 2009 and March 2010 reported that 71.8% of the patients had a CD4 count <350 cells/mm^3^ at HIV diagnosis. A study in USA in 2001 [15] showed that the mean CD4 count in HIV patients was 246 cells/mm^3^, and the median was 152 cells/mm^3^. Therefore, earlier diagnosis of HIV-infected patients is a big challenge for all countries, especially the developing countries.

The main reasons for late diagnosis of HIV infection may be low initiative towards HIV testing and the discrimination and stigma attached to the infection [13]. A late diagnosis may also be due to a relatively weak knowledge among medical staff towards HIV associated diseases [13]. China currently provides free services of VCT for the public. However, because the general public in China still has a limited awareness of HIV/AIDS and their active acceptance of VCT service needs to be improved, most HIV infected patients do not know their HIV status. More sustained and vigorous awareness campaigns still need to be done to diagnose this disease early. Moreover, continuing education in HIV/AIDS among healthcare providers should also be reinforced.

We found that the median CD4 count at HIV diagnosis differed significantly according to age, ethnicity, HIV transmission route and province. Although the reasons for this finding remain unclear, it may reflect various factors including patient characteristics, the medical setting, and factors related to reasons for HIV testing. A study conducted in India [16] showed that there is a considerable variation in CD4 counts in the Indian population, and that there exists regional diversity in the T-lymphocyte subset counts. China is a country with a large territory and an extremely large population. The difference in CD4 counts in newly diagnosed HIV-infected patients may reflect regional diversity in the T-lymphocyte subset counts in China. Our study indicates the need to establish a national reference range for absolute counts in healthy Chinese men and women.

In agreement with other studies [17-19], risk factors for late-stage HIV disease presentation arose in diverse domains of influence, including geographic, economic, demographic, social, and psychosocial. In our sample, males, younger people, people diagnosed in the provinces Guangxi, Henan, Heilongjiang, Jiangxi, Shanghai and Yunnan, and persons who acquired the infection through unknown transmission route showed a higher risk of low CD4 count at HIV diagnosis. These findings provide focused targets for improving HIV testing programs in order to diagnose people earlier and reduce the number of adults presenting to care with late-stage HIV disease. Different strategies and measures should be taken for different regions and populations to detect HIV infection as early as possible.

The finding that patients reported in the provinces of Guangxi, Henan, Heilongjiang, Jiangxi, Shanghai and Yunnan were more likely to have low CD4 count at HIV diagnosis, suggests a poorer access to HIV testing centers or a lower risk perception of HIV infection. Increased efforts should be made to identify new cases of HIV infection in these provinces.

In keeping with other reports [17], males were more likely to be diagnosed later in infection than females, and intravenous drug users were associated with lower risks of late presentation. The lower proportion of females being diagnosed late can be attributed to a higher uptake of VCT services by the females as part of routine health care services during pregnancy [17]. The known high risk of HIV infection in intravenous drug users means that clinicians are often more active in providing HIV testing for this group and this may result in earlier patient detection. Homosexual transmission route has been reported to be associated with a lesser risk of late presentation in previous studies [18,20], this finding may be associated with a higher probability among MSM (men who have sex with men) of undergoing HIV testing which reflects a higher perception of sexual risk behavior. In our study, we could not analyze the association between homosexual or heterosexual transmission route and low CD4 count because the data on homosexual sex were not available. This may lead to ignorance of such important risk factor and subsequently under prioritize intervention in this special group. Our finding that unknown HIV transmission route and younger age were associated with a higher risk of low CD4 count may be associated with a lower probability among these populations of undergoing HIV testing. A study [21] from San Francisco showed that late HIV testing was more likely among persons <30 years old, heterosexuals, and persons without a reported risk.

There are some notable limitations to this study. First, potential sample selection bias may have affected the findings. The HIV epidemic is serious in some areas and among some most-at-risk populations in China, hence the study population is not representative of the entire newly diagnosed HIV positive population in China and so the results may not be generalizable. Second, the design of the study was observational, we were able to examine potential associations but were unable to assess causation. Third, we did not assess some factors (cultural, psychological, socioeconomic) shown to be associated with low CD4 count in other studies [19]. Further studies assessing the role of other factors may help understanding the cause of late HIV diagnosis, and should be undertaken to improve the management of HIV infection in China. Fourth, inaccuracy and incompleteness of documentation in the patients' medical histories, as well as variation in equipment and techniques used by different provinces to analyze the CD4 counts might have decreased the accuracy of our data or introduced biases.

## Conclusions

At the time of HIV diagnosis, a large proportion of HIV-infected patients in China had an initial CD4 count that was consistent with relatively advanced disease. Males, younger people, people diagnosed in provinces Guangxi, Henan, Heilongjiang, Jiangxi, Shanghai and Yunnan, and persons who acquired the infection through unknown transmission route showed a higher risk of low CD4 count at HIV diagnosis. These results reinforce the importance of identifying HIV infection as early as possible and suggest the need for a more active offer of HIV testing in these populations.

## Competing interests

The authors declare that they have no competing interests.

## Authors’ contributions

YZS and HZL conceived of the study, and participated in its design and coordination. YZS performed the statistical analysis and drafted the manuscript. ZYW, TKQ, and JRW participated in data collection. All authors read and approved the final manuscript.

## Pre-publication history

The pre-publication history for this paper can be accessed here:

http://www.biomedcentral.com/1471-2334/13/429/prepub
